# The role of a cuproptosis-related prognostic signature in colon cancer tumor microenvironment and immune responses

**DOI:** 10.3389/fgene.2022.928105

**Published:** 2022-10-12

**Authors:** Chenyang Xu, Yonghao Liu, Yuxi Zhang, Ling Gao

**Affiliations:** ^1^ Department of General Surgery, The First Affiliated Hospital of Soochow University, Suzhou, China; ^2^ Department of Imaging, The First Affiliated Hospital of Soochow University, Suzhou, China

**Keywords:** cuproptosis, colon adenocarcinoma, prognosis, tumor microenvironment, immune responses

## Abstract

**Background:** Colon adenocarcinoma (COAD) is a common malignant tumor of the digestive tract with poor clinical outcomes. Cuproptosis is a novel cell death mechanism and linked to mitochondrial respiration. However, the role of cuproptosis in colon cancer tumor microenvironment (TME) and immune responses remains unknown.

**Methods:** We conducted difference analysis to identify the differential expressed cuproptosis-related genes (CRGs). According to the CRGs, the TCGA-COAD samples were categorized using consensus clustering. The LASSO regression analysis was utilized to develop the cuproptosis-related signature. We then verified the model reliability by Kaplan–Meier, PCA, and ROC analysis. The GES39582 cohort served as the validation set. GO and KEGG functional analyses were conducted to investigate the underlying mechanism. We compared the infiltration levels of immune cells, the expression levels of immune checkpoints, and microsatellite instability (MSI) status between the high- and low-risk groups. Additionally, the relationships between the risk signature and immune cells and cancer stem cell (CSC) were analyzed.

**Results:** Finally, we identified 9 differentially expressed CRGs in COAD. According to the expression of CRGs, the TCGA-COAD samples were separated into two clusters. The 11-gene signature was established by LASSO, and it had excellent predictive power for COAD prognosis. Besides, we used the GSE39582 cohort to validate the prognostic value of the model. GO and KEGG results demonstrated that the survival differences between two risk groups was mainly linked to the extracellular matrix (ECM). Further immune characterization analysis showed the significant differences in the immune cell infiltration and immune responses between two risk groups.

**Conclusion:** Overall, the novel cuproptosis-related signature was able to accurately predict COAD prognosis and played important roles in COAD tumor microenvironment and immune responses.

## Introduction

Colon adenocarcinoma (COAD) is a common malignant tumor of the digestive tract, and it is the third leading cause of cancer-related deaths worldwide (Cassidy and Syed, 2017). Despite advances in surgical and complementary therapy, the clinical outcomes for COAD patients remain poor (Benson et al., 2018; Miller et al., 2019). The condition is caused by the complex pathogenesis of COAD. Most COAD patients are in advanced stage when diagnosed. Therefore, it is urgent to find excellent biomarkers for COAD diagnosis and prognostic prediction.

Copper-dependent death called cuproptosis was recently reported, which is induced by direct binding of copper to lipoylated components of the tricarboxylic acid (TCA) cycle (Tsvetkov et al., 2022). The studies revealed that the killing effect of copper carrier on cells is likely related to the process of mitochondrial respiration. Remarkably, a number of key genes promoting copper death were identified in the previous studies, including FDX1 gene encoding elesclomol molecular target protein, and several genes involved in mitochondrial metabolism and protein acylation modification (Kahlson and Dixon, 2022). Acylation modification is a chemical modification of proteins in mitochondria, which plays a critical role in mitochondrial metabolism (Tan et al., 2014; Hirschey and Zhao, 2015). Copper ions in mitochondria directly bind to fatty acylation modified proteins through copper carrier, resulting in their formation of long chains and agglomeration and cell death. These copper ions also interfere with iron sulfur clusters, resulting in down-regulation of iron sulfur protein, which in turn leads to cytotoxic stress and death (Kahlson and Dixon, 2022).

Currently available researches have clearly demonstrated that cuproptosis plays a critical role in tumor progression. Nevertheless, the specific role of cuproptosis in COAD has never been studied. Thus, we conducted a comprehensive analysis to explore the expression level of the cuproptosis-related genes (CRGs) between normal and cancerous samples, evaluate the prognostic value of the cuproptosis-related signature, and explore the relationship between cuproptosis and tumor microenvironment (TME).

## Materials and methods

### Data collection

The flowchart of this work was present in the Supplementary Figure S1. The RNA sequencing data (41 normal samples and 480 tumor samples) and corresponding clinical information for COAD samples were obtained from the TCGA database (https://portal.gdc.cancer.gov/repository). The data downloaded from the GEO database (https://www.ncbi.nlm.nih.gov/geo/, ID: GSE39582) served as the external validation cohort. Samples with missing clinical data were excluded.

### Identifcation of diferentially expressed CRGs

13 cuproptosis-related genes (CRGs) were extracted from the previous study, including FDX1, LIPT1, LIAS, DLD, DBT, GCSH, DLST, DLAT, PDHA1, PDHB, SLC31A1, ATP7A, and ATP7B (Tsvetkov et al., 2022). The diferentially expressed CRGs with the *p*-value < 0.05 were identified *via* the “limma” package. For investigating the interaction of CRGs,a PPI network was developed through the STRING database.

### Consensus clustering

Consensus clustering was performed to identify distinct cuproptosis-related subtypes associated with the expression of the CRGs by the k-means method. The overall survival (OS) of patients in the two subtyps was compared by Kaplan–Meier (KM) survival analysis. OS was defined as the length of time from the data of diagnosis to date of death or last follow-up time. In addition, we screened the differentially expressed genes (DEGs) betwween the two clusters obtained from the cluster analysis for further research. These DEGs were selected as candidate genes for model development.

### Development and verification of the risk signature

We performed Cox regression analysis for assessing the prognosis of the DEGs between the two clusters in the TCGA cohort. The *p*-value < 0.01 was set as the cut-off criterion, and 27 OS-related genes were selected for subsequent analysis. For avoiding overfitting of the model, LASSO regression analysis was performed with the R package “glmnet”. The risk score of the patients was calculated according to the normalized expression level of each gene and corresponding regression coefficient as the following formula:

$$RiskScore=\overset{N}{\underset{i=1}{\sum }}\left(Expi\times Coei\right).$$ (1)

Furthermore, the samples were separated into high-risk and low-risk groups according to the median risk score. The OS of COAD patients in two groups was compared by KM survival analysis. Besides, PCA analysis was used to assess sample clustering, and ROC curves were perform for evaluating the model predictive capability. The independence of the risk model was validated by univariate and multivariate cox analysis. Finally, the reliability of the model was confirmed by the validation cohort (GSE39582). For better clinical application of the risk model, a nomogram for 1-, 3-, 5-year OS was developed, and its reliability was validated by calibration curves.

### Functional enrichment and immune characterization analysis

The TCGA-COAD cohort was classified into two subgroups based on the median risk score. A cutoff of |log2FC| ≥ 0.585 and FDR <0.05 was used to screen DEGs between the two subgroups. GO and KEGG functional analysis based on the DEGs were performed by the “clusterProfiler” R package. The “gsva” package was used for conducting the ssGSEA to calculate the scores of immune cells and to assess the activity of immune-related pathways. We quantified the abundance of 22 immune cells by CIBERSORT and analyzed the relationship between immune cells and 11 genes. Furthermore, the expression levels of immune checkpoints between two risk groups were compared by boxplots. Finally, we explored the correlations of risk score and microsatellite instability (MSI) along with cancer stem cell (CSC). We integrated transcription gene expression data with stemness score (RNAss) to perform the Spearman correlation analysis.

### Statistical analysis

Statistics analysis was conducted by R software (V4.1.0). Pearson chi-square test was applied to analyze the categorical variables. For comparing the OS of different subgroups, KM survivial curves was conducted with a two-sided log-rank test. Univariate and multivariate Cox regression analysis were used to evaluate the independence of the risk model. We evaluated immune cell infiltration and immune pathway activation in the two risk groups by Mann-Whitney test. *p* values were showed as: *p < 0.05; **p < 0.01; ***p < 0.001. *p* < 0.05 was considered significant.

## Results

### Identification of cuproptosis-related DEGs

**FIGURE 1:**
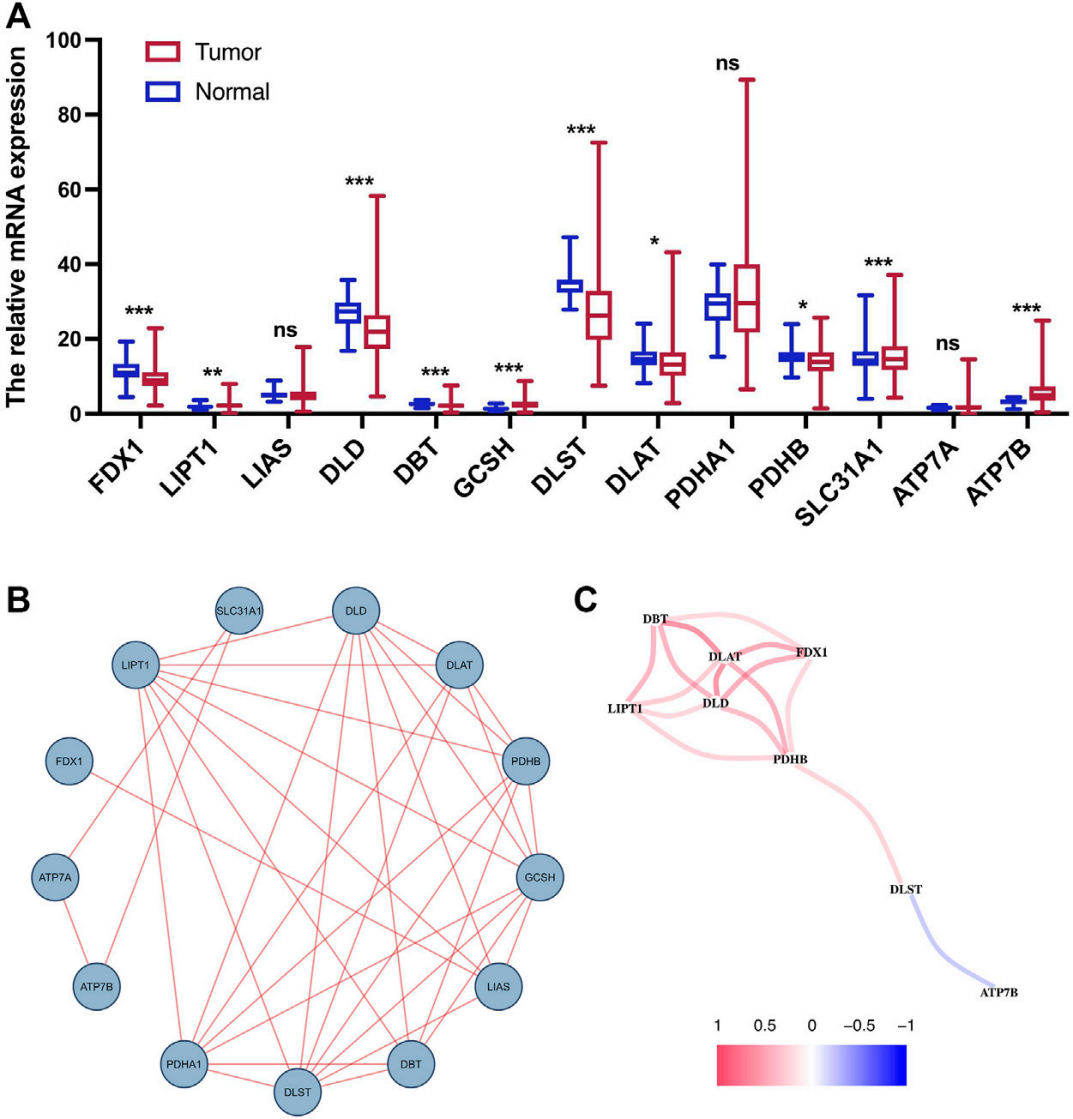
Expression and interaction of CRGs. **(A)** Heatmap of cuproptosis-related DEGs between normal tissue and tumor tissue. **(B)** PPI network of 13 CRGs. **(C)** Correlation network of cuproptosis-related DEGs. ns, *p* > 0.05. ns, not significant; *, *p* < 0.05; **, *p* < 0.01; ***, *p* < 0.001.

We performed differential expression analysis on 13 CRGs. The heatmap indicated that nine CRGs were screened out as DEGs, of which six cuproptosis-related DEGs were lowly expressed in tumor tissues and the other three were upregulated (Figure 1A). For further understanding the interactions among the CRGs, a PPI network was shown in Figure 1B. In addition, the correlation coefficients between the cuproptosis-related DEGs were calculated, and the result revealed a significantly positive correlation between the DEGs, except for ATP7B (Figure 1C).

### Classification of COAD patients based on the DEGs

**FIGURE 2:**
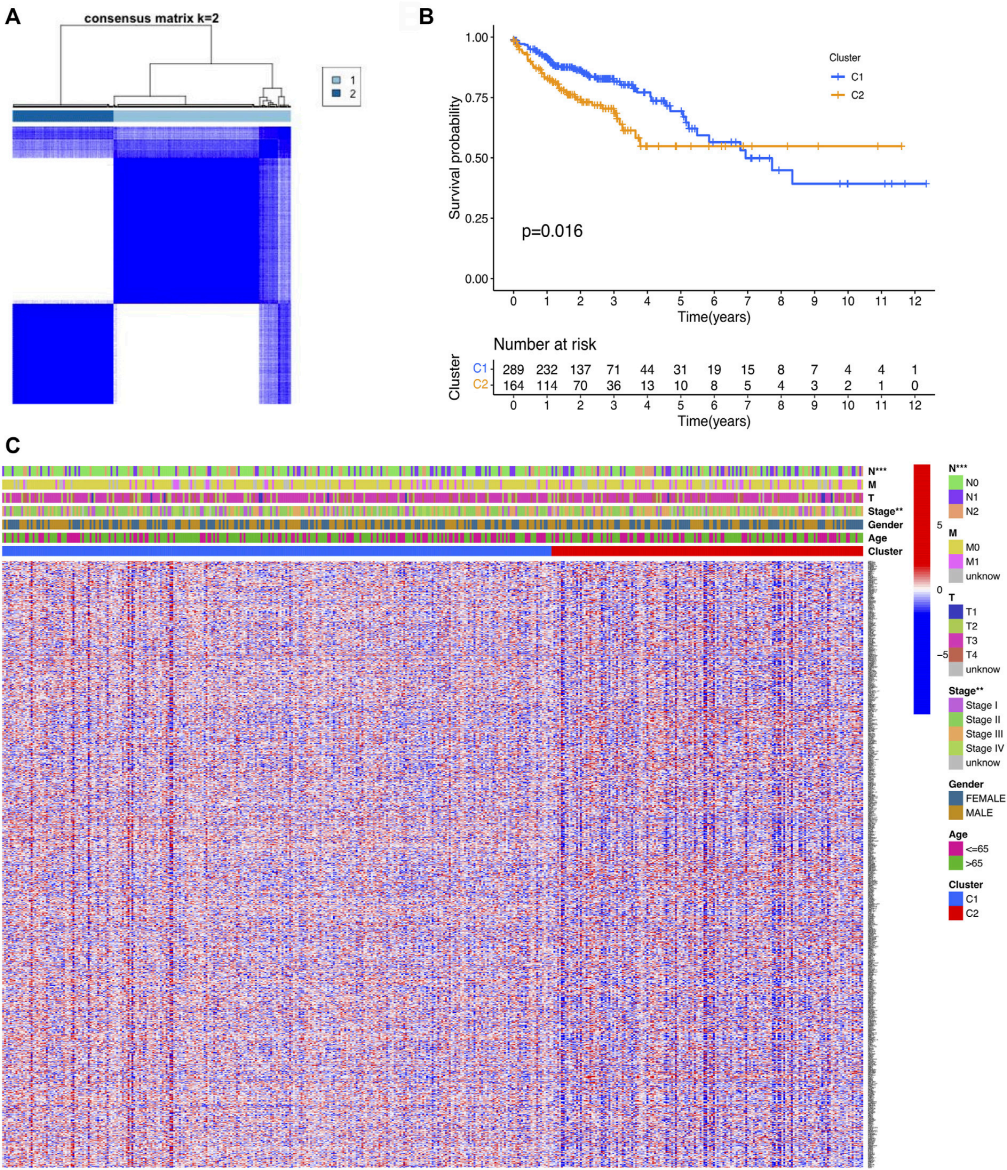
Classification of COAD patients based on the cuproptosis-related DEGs. **(A)** Total of 521 COAD patients were clustered into two clusters according to the consensus clustering matrix (k = 2). **(B)** KM curve for the two clusters. **(C)** Heatmap and the clinicopathologic features of the two clusters classified by the cuproptosis-related genes.

On the basis of the cuproptosis-related DEGs, two clusters were identified by the consensus clustering analysis (Figure 2A). We compared the survival curves between the two clusters and found that the patients in cluster 1 had better survival than these in cluster 2 (Figure 2B). For further investigating the distinction between the two clusters, the DEGs of the two clusters were identified. Next, we generated a heatmap comprising gene expression level and clinical features. The heatmap showed that the two clusters had a significant difference in tumor stage and T stage (Figure 2C).

### Establishment and validation of a prognostic risk signature

**FIGURE 3:**
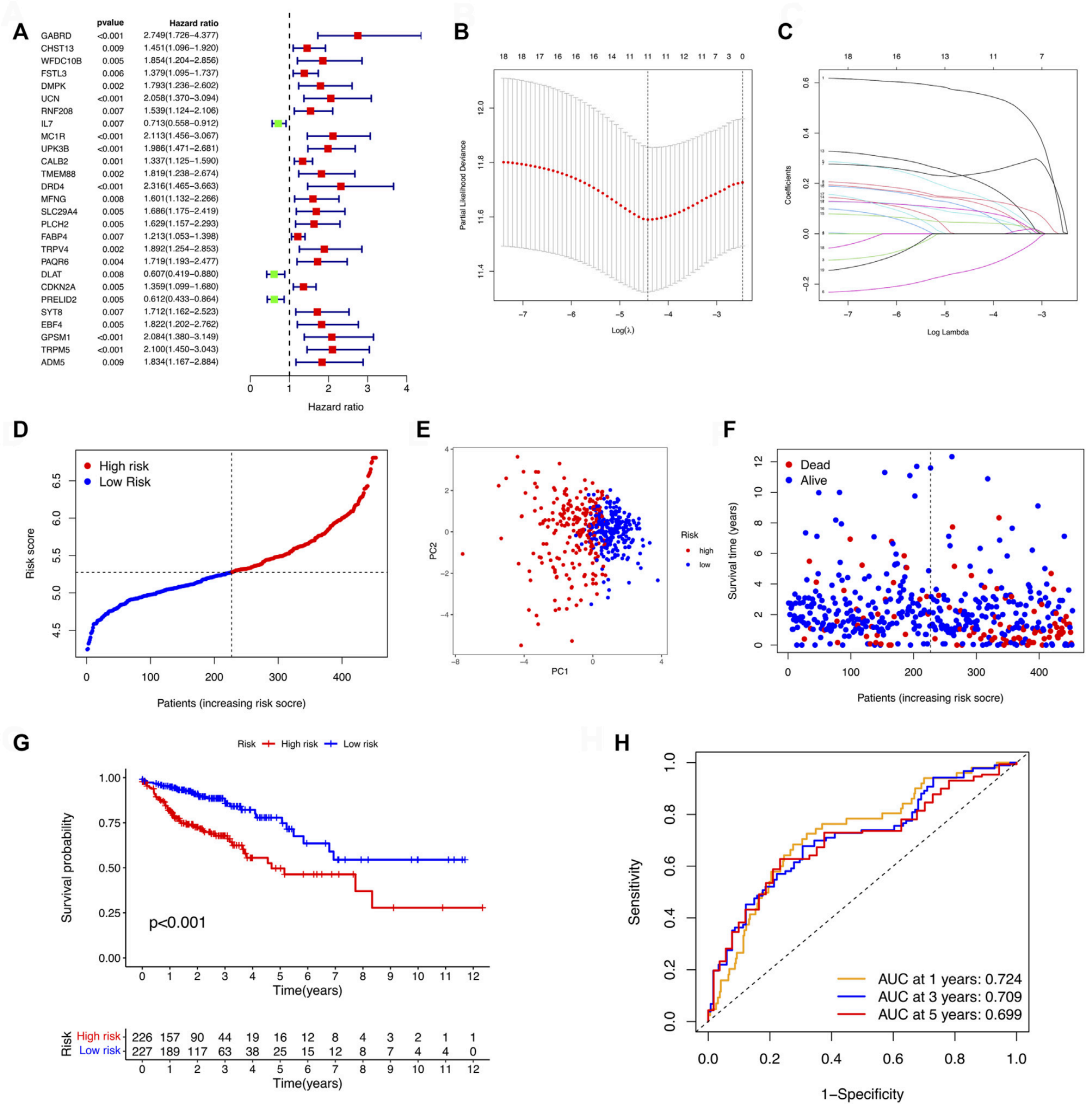
Establishment of the cuproptosis-related signature. **(A)** Forest plots revealing the univariate Cox regression analysis result for the OS of the DEGs between the two clusters. **(B)** Cross-validation for the optimal parameter and operator model selection in the LASSO regression. **(C)** LASSO regression of the 27 prognosis-related genes. **(D)** Distribution of patients based on the risk score. **(E)** PCA plot for COAD upon the basis of risk score. **(F)** Survival status of COAD patients. **(G)** KM curves for the OS of patients in the two risk groups. **(H)** ROC curves manifested the prognostic performance of the risk score in the COAD cohort. AUC: Area under the curve.

**FIGURE 4:**
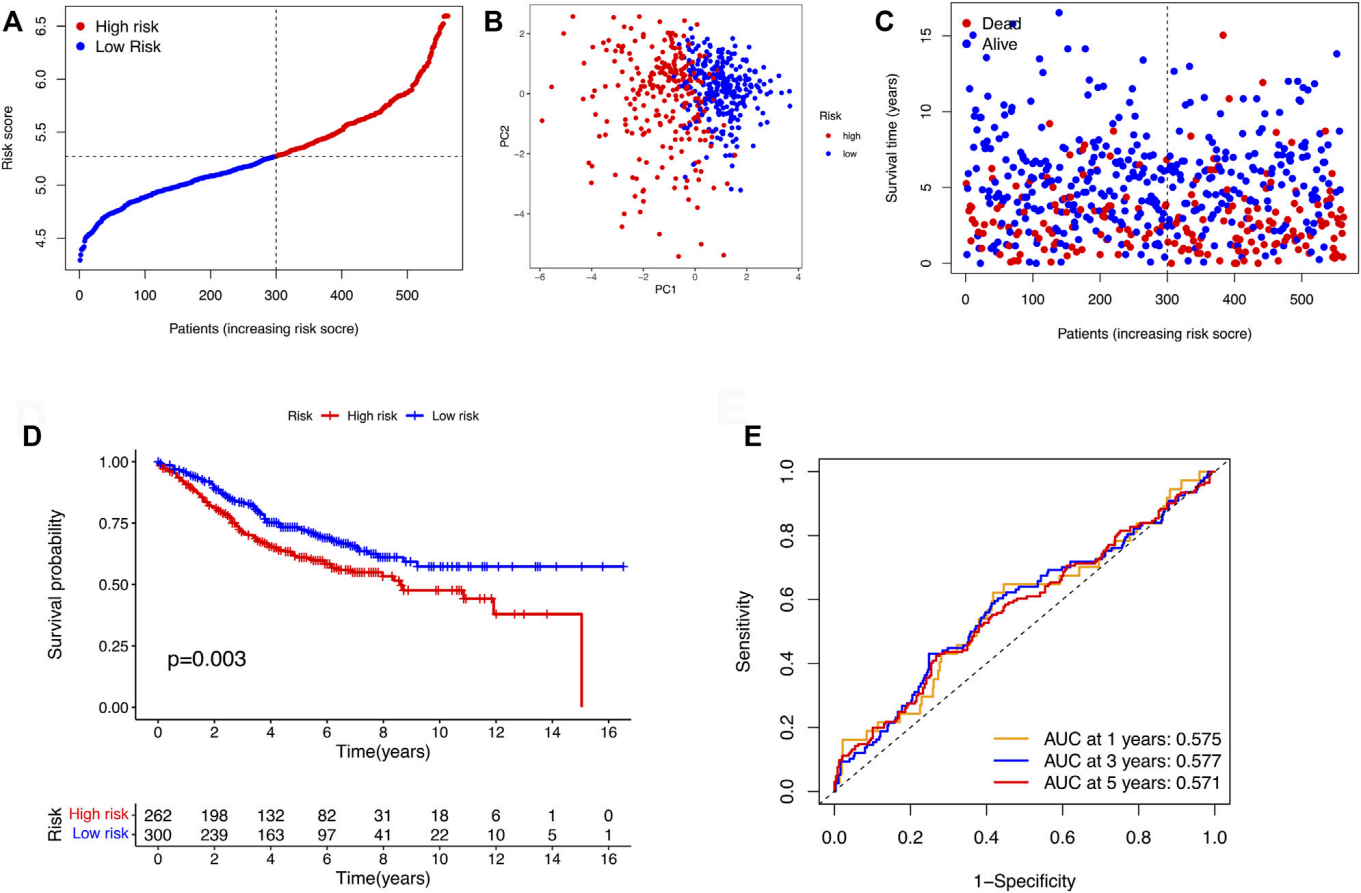
Validation of the risk models in the GSE39582 cohort. **(A)** Distribution of samples in the GSE39582 cohort based on the median risk score in the TCGA cohort. **(B)** PCA plot of the GSE39582 cohort. **(C)** The survival status for COAD patients. **(D)** KM survival curve for the OS of patients between the two risk groups in the GSE39582 cohort. **(E)** ROC curves verified the prognostic performance of the model in the GSE39582 cohort.

After the DEGs between the two clusters were obtain, we performed univariate Cox regression analysis to identify prognostic-related genes. The 27 genes that met the criteria of *p* < 0.01 were screened for subsequent research (Figure 3A). To avoid over-fitting of the risk signature, LASSO regression was performed to screen 11 genes for the model construction (Figures 3B,C). The formula for the model was as follows: Risk Score = (0.562 × GABRD exp.) + (0.126 × CHST13 exp.) + (0.157 × RNF208 exp.) + (−0.143 × IL7 exp.) + (0.237 × MC1R exp.) + (0.139 × CALB2 exp.) + (0.097 × MFNG exp.) + (0.076 × PLCH2 exp.) + (0.092 × FABP4 exp.) + (0.217 × PAQR6 exp.) + (0.044 × CDKN2A exp.). On the basis of the medium risk score, we separated the samples into the high- and low-risk groups (Figure 3D). The principal component analysis (PCA) result exhibited a clear grouping (Figure 3E). Compared with the samples in the low-risk group, those in the high-risk group showed more deaths and less survival times (Figure 3F). The overall survival (OS) between two risk groups exhibited a significant difference (*p* < 0.001, Figure 3G). To determine the model predictive efficacy, the ROC curves was conducted to show the AUC for the 1-year, 2-year, and 3-year survival. Their values were 0.724, 0709, and 0.699 respectively, indicating the excellent predictive power of the model. In addition, for validating the prognostic value of the model, the GSE39582 cohort was utilized as the validation cohort. Not surprisingly, the predictive performance of the risk signature in the GSE39582 cohort was consistent with that in the TCGA cohort (Figure 4).

### Independent prognostic value of the cuproptosis-related signature

**FIGURE 5:**
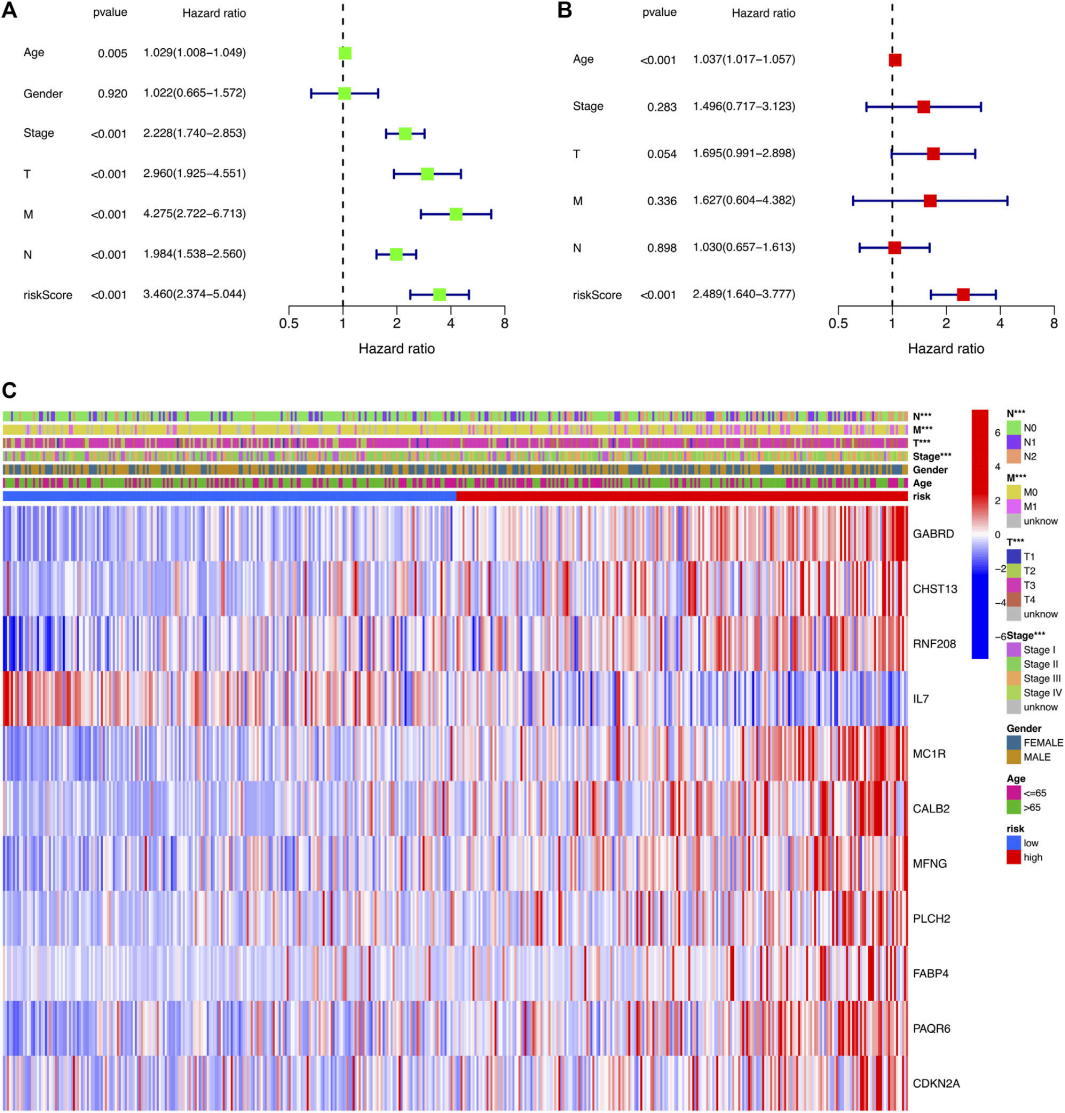
Independent prognostic analysis of the risk signature. **(A)** Univariate analysis for the TCGA-COAD cohort. **(B)** Multivariate analysis for the TCGA-COAD cohort. **(C)** Heatmap exhibiting the correlation of clinical characteristics and the risk groups.

**FIGURE 6:**
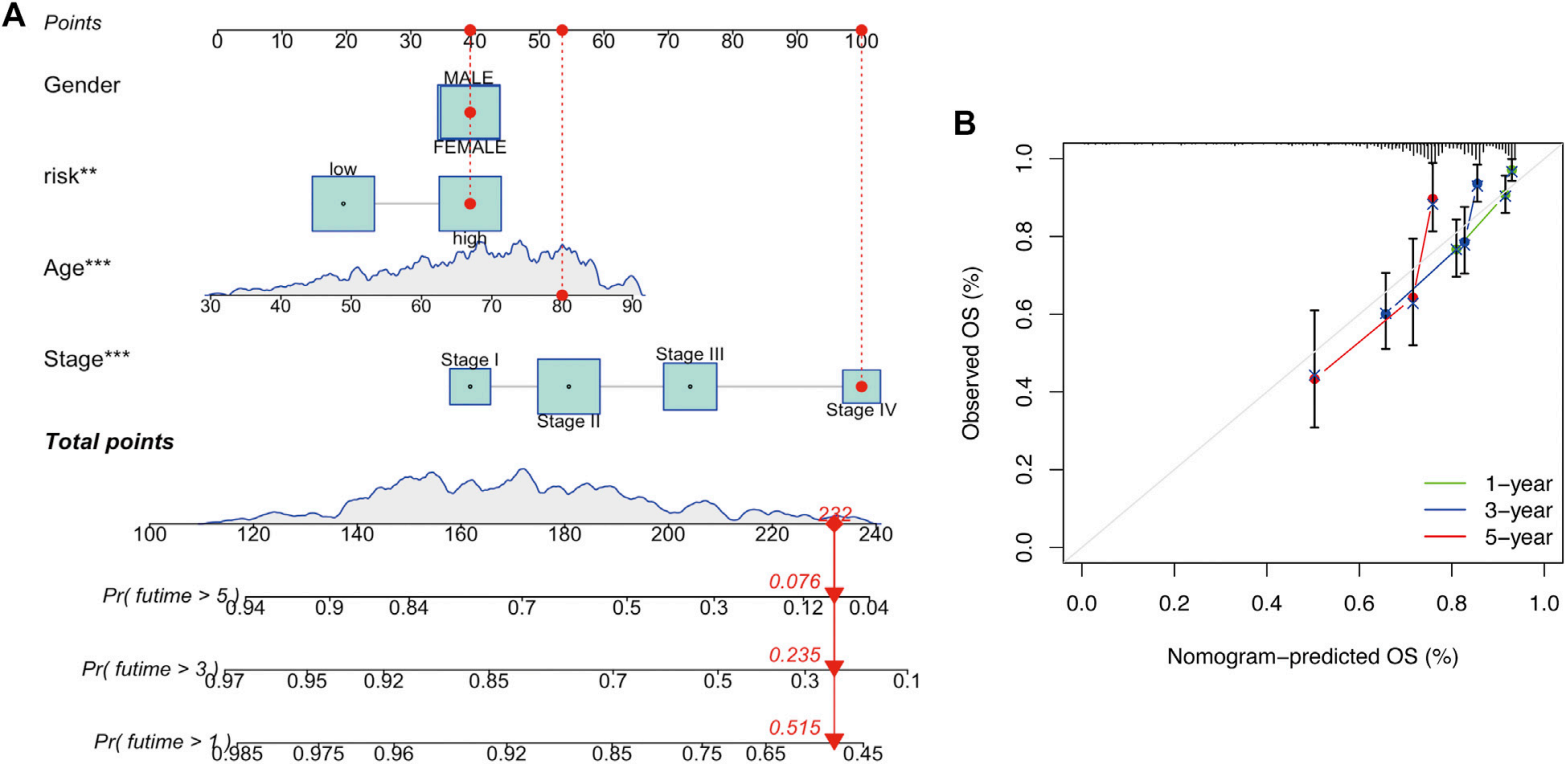
Development and verification of a nomogram. **(A)** Nomogram for one-, three-, and 5-year OS. **(B)** Calibration curves.

To determine whether risk score could serve as an independent prognostic factor for COAD, univariate and multivariable Cox regression analysis were performed. The results indicated that the risk signature could be independent of other clinical features (Univariate Cox regression: *p* < 0.001, HR = 3.460, 95% CI = 2.374–5.044; Multivariable Cox regression: *p* < 0.001, HR = 2.489, 95% CI = 1.640–3.777, Figures 5A,B). Besides, a heatmap containing clinical features and gene expression level was presented in Figure 5C, from which we found that tumor stage, T stage, N stage and M stage revealed significant differences in two risk groups (Figure 5C). The nomogram was showed in Figure 6A. The calibration curves suggested that the excellent agreement between the predictions and actual outcomes (Figure 6B).

### Functional enrichment analysis

**FIGURE 7:**
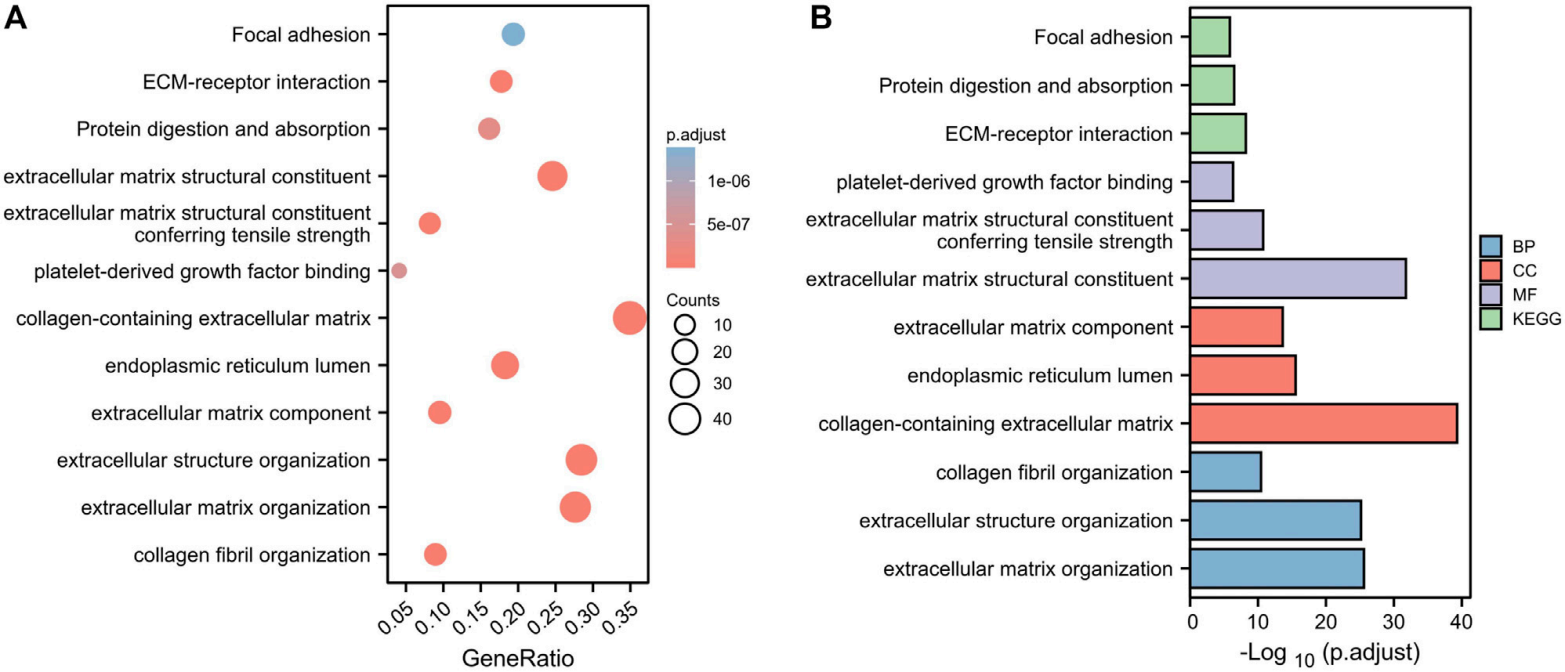
Functional analysis based on the DEGs between the two risk groups in the TCGA cohort. **(A)** Bubble diagram of GO and KEGG enrichment (the bigger bubble means the more genes enriched, and the increasing depth of red means the differences were more obvious). **(B)** Bar plot graph of GO and KEGG enrichment (the longer bar means the more genes enriched, and the increasing depth of red means the differences were more obvious).

To investigate the potential molecular mechanisms of the risk signature, we performed GO and KEGG functional analysis on the DEGs between the high- and low-risk groups. The results demonstrated that the DEGs were mainly related to extracellular matrix (ECM, Figures 7A,B).

### Immune characterizations analysis

**FIGURE 8:**
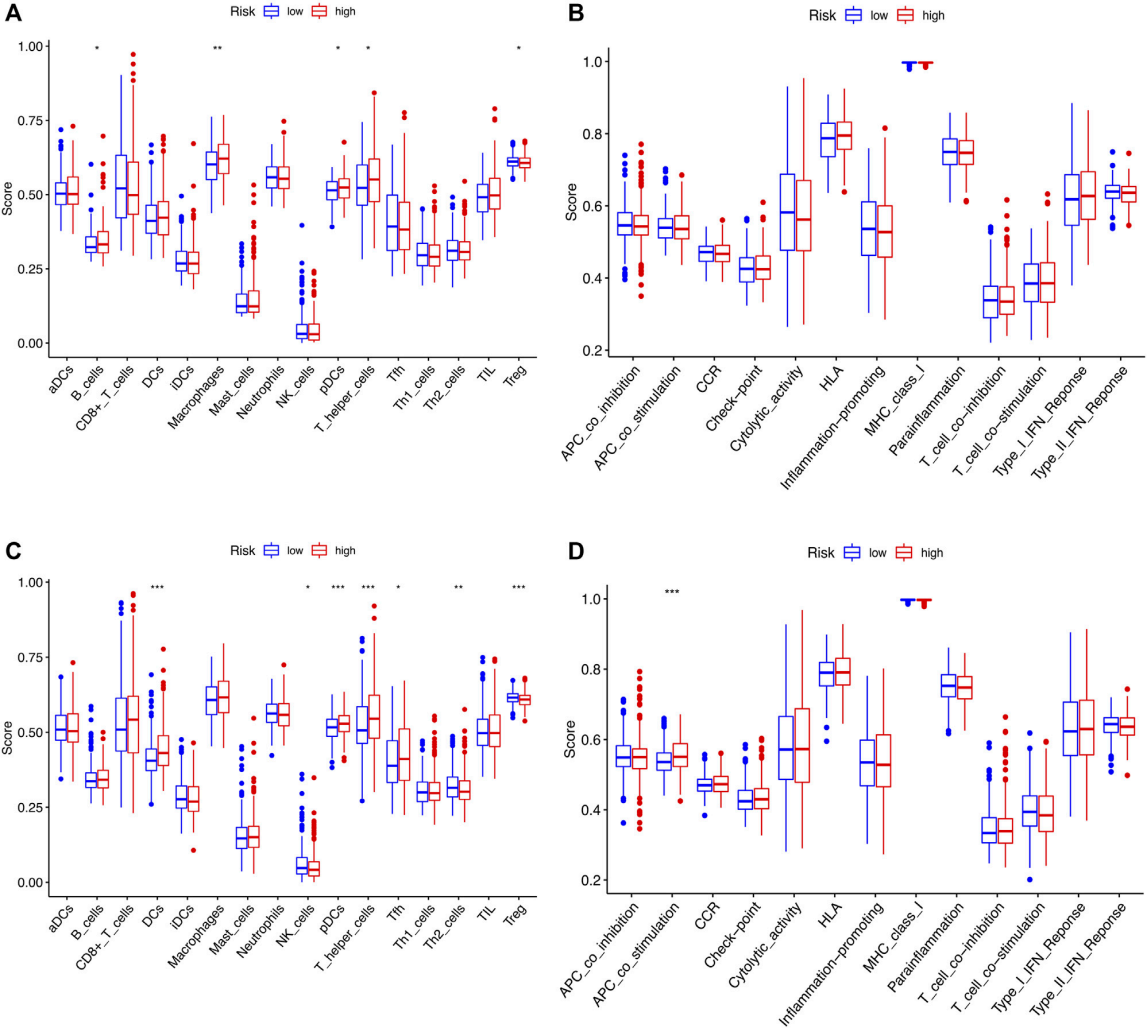
Comparison of ssGSEA scores for immune cells and immune pathways between two risk groups in the TCGA cohort and GSE39582 cohort. **(A and B)** The enrichment scores of 16 types of immune cells and 13 immune-related pathways between the two risk groups in the TCGA cohort. **(C,D)** Immune characterizations between the two risk subgroups in the GEO set. ns, *p* > 0.05. ns, not significant; *, *p* < 0.05; **, *p* < 0.01; ***, *p* < 0.001.

**FIGURE 9:**
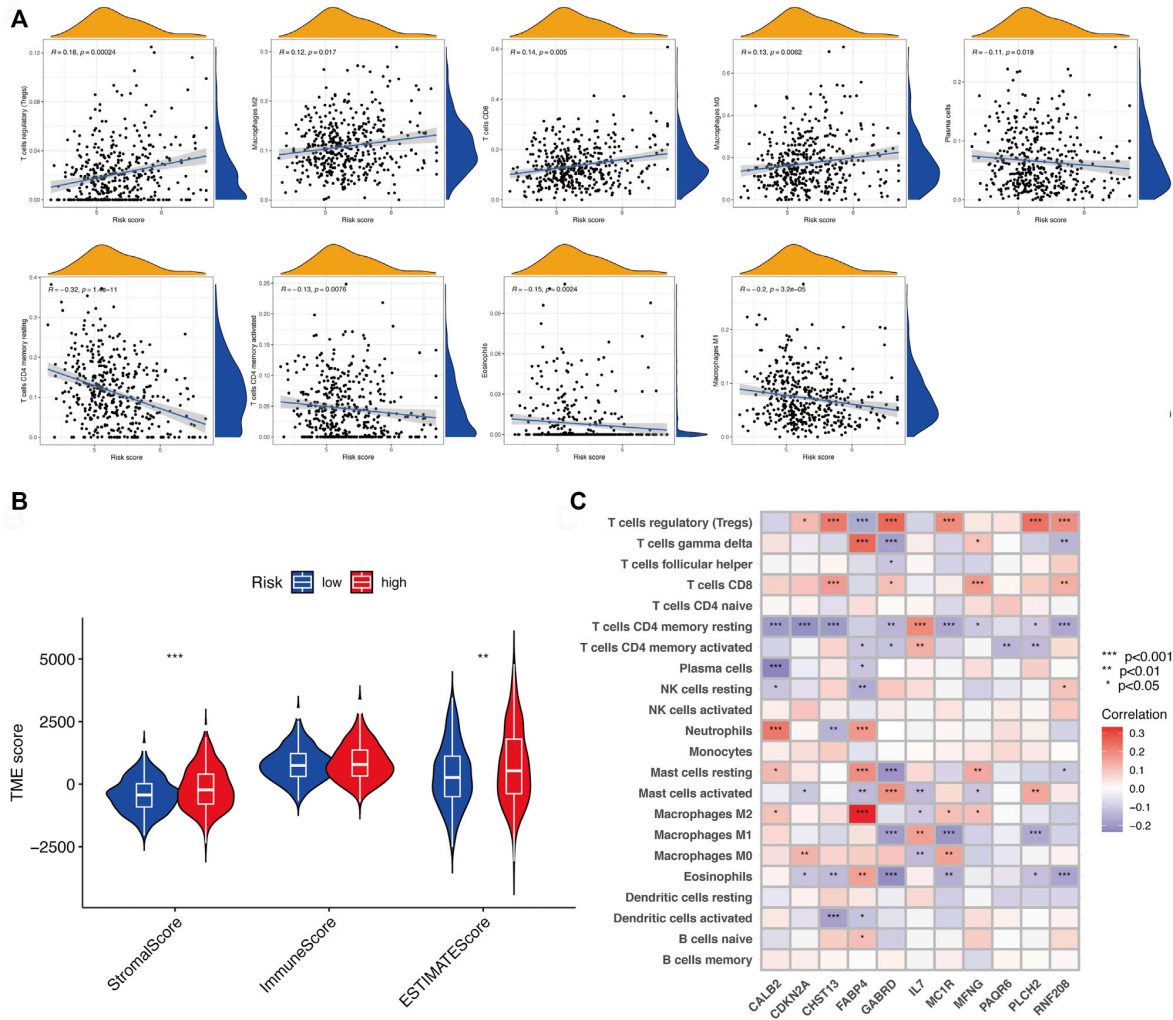
Assessment of TME between two risk subgroups. **(A)** Correlation of risk score and different types of immune cells. **(B)** Immune and stromal scores in the risk groups. **(C)** Correlation between 11 genes and 22 types of immune cells. ns, *p* > 0.05. ns, not significant; *, *p* < 0.05; **, *p* < 0.01; ***, *p* < 0.001.

**FIGURE 10:**
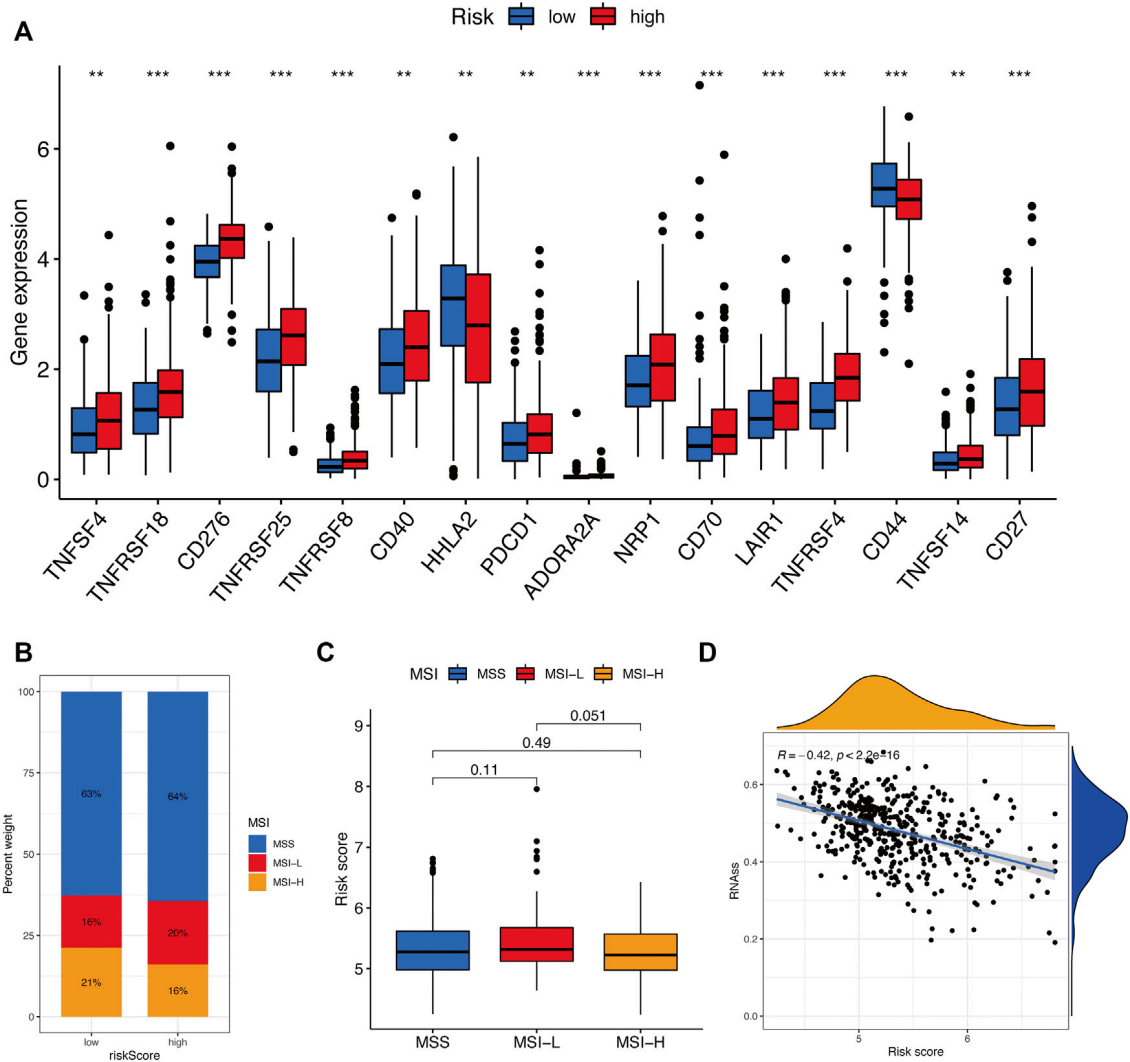
Evaluation of the immunotherapy response between two risk groups. **(A)** Immune checkpoint expression levels between the high- and low-risk groups. **(B and C)** Correlation between risk score and MSI. **(D)** Correlation between risk score along with CSC index. ns, *p* > 0.05. ns, not significant; *, *p* < 0.05; **, *p* < 0.01; ***, *p* < 0.001.

The tumor microenvironment (TME) consists of tumor cells, blood vessels, immune cells, stromal cells and ECM (Kessenbrock, Plaks and Werb, 2010). According to the GO and KEGG results, we found that the risk signature was closely related to the ECM and then further explored the differences between two risk groups in immune cell infiltration and immune-related pathways. In the TCGA and GEO cohorts, the high-risk groups showed higher infiltration levels of plasmacytoid dendritic cells (pDCs) and T helper cells than the low-risk group. The reverse was observed for the regulatory T cells (Treg, Figures 8A,C). However, no significant differences were evident in immune-related pathways between the high- and low-risk groups (Figures 8B,D). To investigate the correlation between the risk signature and the abundance of immune cells, the CIBERSORT algorithm was used for further analysis. As shown in Figure 9A, the risk score was positively correlated with regulatory T cells (Tregs), CD8 + T cells, M0 and M2 macrophages, whereas it was negatively correlated with plasma cells, M1 macrophages, eosinophils, activated and resting memory CD4 + T cells. A high risk score showed a high stromal score and a high ESTIMATE score (Figure 9B). Except for naive CD4 + T cells, activated NK cells, and memory B cells, all immune cells had a significant correlation with at least one of 11 genes (Figure 9C). In addition, we evaluated the correlation between immune checkpoints and the risk signature. Surprisingly, all 16 immune checkpoints exhibited differential expression between the high- and low-risk groups (Figure 10A). It has been reported that high microsatellite instability (MSI-H) represented the greater sensitivity to immunotherapy (Ganesh et al., 2019). Figure 10B indicated that the number of patients with MSI-H status in the low-risk group was larger than that in the high-risk group. Meanwhile, a low risk score was associated with MSI-H status (Figure 10C). Finally, correlation analysis showed that risk score had a significant negative correlation with the CSC index (R = −0.42, *p* = 2.2e-16), indicating that tumor cells with a low risk score had a lower degree of cell differentiation (Figure 10D).

## Discussion

Colon cancer is a common malignancy caused by the progressive accumulation of genetic and epigenetic changes, which induce the transformation from normal epithelium into adenocarcinomas (Kinzler and Vogelstein, 1996; Fodde, 2002). COAD is the most common type of colon cancer. Due to the molecular pathogenesis complexity, the clinical outcomes of COAD patients are consistently poor (Jones et al., 2017). In the past few years, a great deal of clinical parameters such as age, sex, histological grade, pathological characteristics and some serum markers have been widely used for COAD prognostic prediction (Verbeke et al., 2011; Oliveira et al., 2018). However, it is far from enough to develop individualized treatment strategies based on these single factors.

Recent studies showed that cuproptosis induces a unique type of cell death and this novel cell death mechanism may suggest new ways for cancer therapy (Tsvetkov et al., 2022). Although the molecular mechanisms of cuproptosis has been elucidated and numerous key genes have been identified, the correlation of CRGs with the OS of COAD patients remains largely ambiguous. Therefore, a systematic analysis was conducted to investigate the potential role of cuproptosis in the prognostic prediction for COAD patients.

In the study, we firstly collected 13 CRGs from the previous research and studied their expression level between normal and cancerous tissues. Since the current study on the cuproptosis is very few, we just selected 13 CRGs to be the candidate. Relatively few candidate genes could affect the precision of parameter estimates of the predictive models. Nevertheless, to ensure the predictive accuracy of the prognostic model, we collected a large number of COAD samples from the TCGA and GEO database (TCGA: 521 samples; GEO: 585 samples). A larger sample size could lead to higher model accuracy. In the following study, cluster analysis was conducted to identify two clusters on the basis of differentially expressed CRGs, which revealed a significant survival difference between cluster 1 and 2. For further exploring the prognostic value of cuproptosis in COAD, we identified DEGs between two clusters and establish the risk model based on these DEGs by univariate and LASSO Cox regression analysis.

Here, the cuproptosis-related signature was composed of 11 genes, including GABRD, CHST13, RNF208, IL7, MC1R, CALB2, MFNG, PLCH2, FABP4, PAQR6, and CDKN2A. Five of these genes was first identified as the prognosis-related molecules of COAD, such as CHST13, RNF208, MFNG, PLCH2, and PAQR6. Except for these five genes, the remaining 6 genes have been reported previously to be related to colon cancer. For instance, IL-7, a pleiotropic cytokine, has been proved to be associated with lymph node involvement and tumor location of CRC (Krzystek-Korpacka et al., 2017). Lymph node metastasis is the most common pathway of metastasis in CRC (Nakai et al., 2017). In addition, CDKN2A can promote CRC cell metastasis by inducing epithelial-mesenchymal transition (EMT) (Shi et al., 2022). High GABRD expression promotes CRC metastasis and poor prognosis. Meanwhile, the expression of GABRD showed a positive correlation with several EMT-related genes (Niu et al., 2020). It has previously been suggested that EMT plays a critical role in tumor invasion and metastasis (Yang and Weinberg, 2008). The EMT program can be triggered by the imbalanced ECM (Schedin and Keely, 2011). From these views, the GO and KEGG results in our study were in accordance with the previous research showing the important roles of ECM in colon cancer. Notably, MC1R and FABP4 were recently identified as prognosis-related immune genes in COAD that associated with immune cell infiltration (Chen et al., 2020; Miao et al., 2020). According to the immune characterizations analysis, we found that the risk signature appeared to be closely correlated with immune cell and stromal infiltration. Immune cells and ECM are critical components of TME (Pietras and Östman, 2010; Hale et al., 2013). Therefore, we speculate that the cuproptosis-related risk signature may play a key role in the TME of COAD. The contribution of the TME to cancer progression has been widely acknowledged in recent years (Lewis and Pollard, 2006; Cacho-Díaz et al., 2020). In the study, we found that the risk score of the cuproptosis-related signature was negatively with CSC index, indicating the tumor cells with high risk score showed stronger proliferation and differentiation capacity.

Immunotherapy is a promising strategies for cancer treatment, and its therapeutic efficacy depends on the interactions between TME and tumors (Kaymak et al., 2021). Among various immunotherapy approaches, immune checkpoint blocking therapy is currently the most developed immunotherapy in clinical applications (Qi et al., 2020). In our study, the expression levels of 16 immune checkpoints showed significant difference between the two risk groups. In addition, it was showed that MSI-H status represented the greater sensitivity to immunotherapy (Ganesh et al., 2019). Therefore, we explored the proportion of patients with MSI-H status in the two risk groups and the correlation of MSI status with risk score. The results showed that patients in the high-risk group were more sensitive to immunotherapy. Overall, the cuproptosis-related risk signature may play a key role in the TME and immune responses of COAD. Future research should aim to explore the specific functions and mechanisms between cuproptosis and TME and immune responses.

Of course, our research has several limitations to be acknowledged. First of all, more prospective clinical data sets are needed to validate the clinical utility of the risk signature. Second, the specific roles and molecular mechanisms of the cuproptosis-related signature in COAD should be validated *in vivo* and *in vitro* experiments. In addition, apart from ECM and immune cell infiltration, the other components of TME need to be further investigated by performing combination analysis with the risk signature.

## Conclusion

Overall, we established a reliable cuproptosis-related signature for the COAD prognostic prediction. The risk signature was linked to immune cell infiltration and CSC index. The underlying mechanism of the risk signature in COAD was the dysregulation of ECM homeostasis. We provide the theoretical basis for further study on the roles of cuproptosis in colon cancer TME and immune responses.

## Data Availability

The datasets presented in this study can be found in online repositories. The names of the repository/repositories and accession numbers can be found in the article/Supplementary Material.
