# Supplementary figures and images for: The role of a cuproptosis-related prognostic signature in colon cancer tumor microenvironment and immune responses
Source: Front Genet. 2022 Oct 12;13:928105. doi: 10.3389/fgene.2022.928105 (PMC9596916; doi:10.3389/fgene.2022.928105)

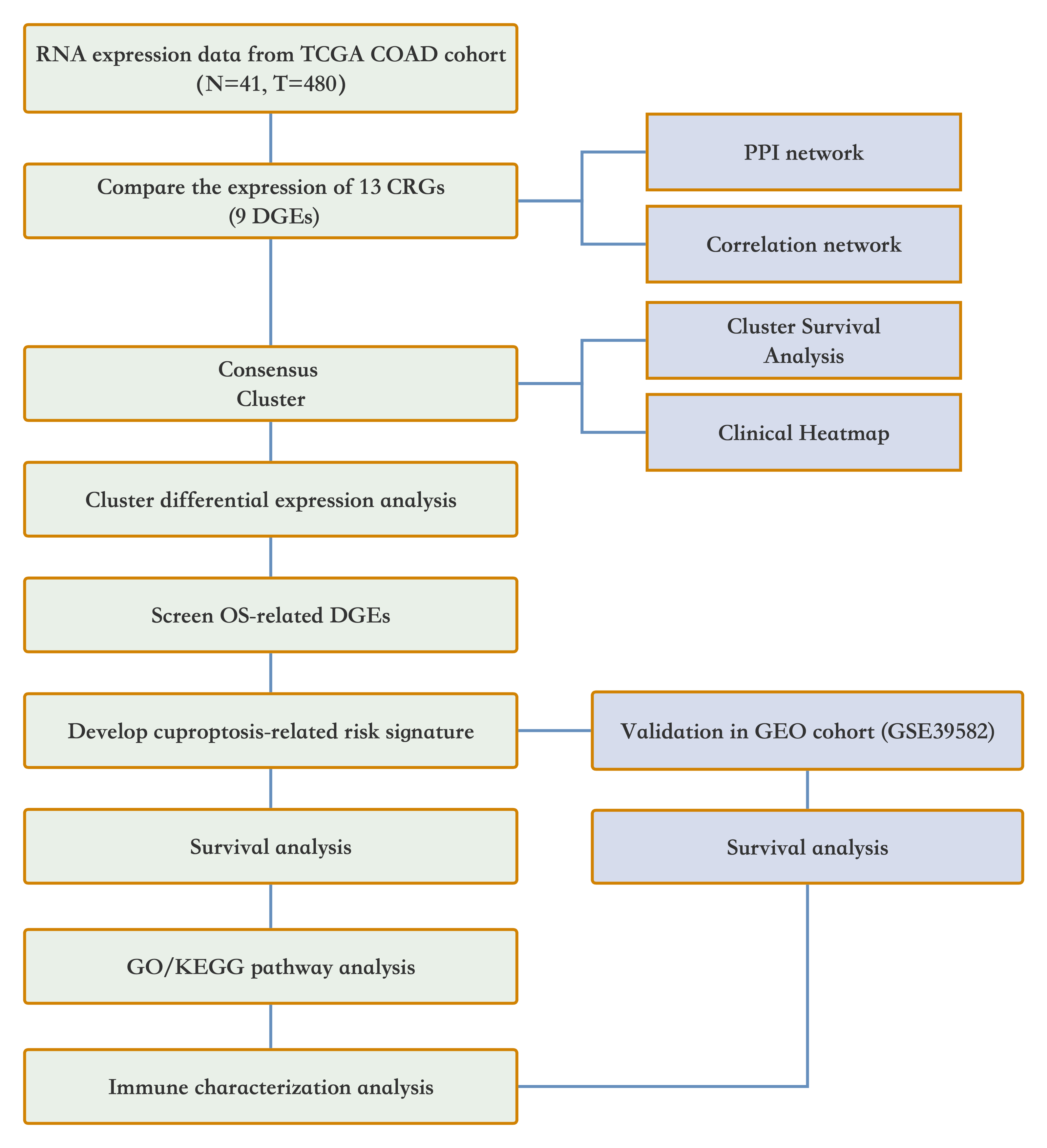

Supplement: Supplementary file 1 [file Image1.JPEG]
